# Adapting *PICU UP!* to Enhance Early Mobility in a Level IV Neonatal Intensive Care Unit: A Quality Improvement Project

**DOI:** 10.1097/pq9.0000000000000862

**Published:** 2026-02-23

**Authors:** Brooke A. Krbec, Denise Casey, Benjamin G. Ethier, Anthony Dekermanji, Megan Dakhlian, Mary-Jeanne Manning, Sapna R. Kudchadkar, Kristen T. Leeman

**Affiliations:** From the * Division of Newborn Medicine, Department of Pediatrics, Boston Children’s Hospital, Harvard Medical School, Boston, Mass.; † Division of Newborn Medicine, Tufts Medical Center, Tufts University School of Medicine, Boston, Mass.; ‡ Department of Pediatrics, Boston Children’s Hospital, Harvard Medical School, Boston, Mass.; § Departments of Anesthesiology and Critical Care Medicine, Pediatrics, and Physical Medicine & Rehabilitation, Johns Hopkins University School of Medicine, Baltimore, Md.

## Abstract

**Introduction:**

Patient acuity in the neonatal intensive care unit can lead to inadequate focus on developmental care. Structured approaches to promote early mobility can improve outcomes.

**Methods:**

Our team adapted and implemented the *PICU Up!* program for the neonatal population and established an early mobility strategy for surgical infants born at 34 weeks’ gestation or later. Our specific aim was to increase the percentage of eligible surgical patients who receive physical therapy (PT) and occupational therapy (OT) consultations from 25% and 22% to greater than 75% within 24 months. Additional measures included time to consult placement, length of stay, pressure injury rate, unplanned extubations, and fractures. The interventions tested a quality improvement framework and Plan-Do-Study-Act cycles, which included the addition of prompts on rounds, education, and documentation optimization.

**Results:**

Control chart analysis showed that the percent of surgical admissions with PT consults increased significantly from 25% to 95% and OT consults from 22% to 95% after project initiation. Time to consultation decreased significantly from 23 to 8 days for PT and from 23 to 9 days for OT consults. There was no significant difference in length of stay, time to first extubation, number of pressure injuries, fractures, or unplanned extubations.

**Conclusions:**

The adaptation and implementation of a standardized approach to early mobility in neonatal intensive care unit patients resulted in increased and more timely PT and OT consultations, leading to an overall improved focus on developmental care with minimal risks.

## INTRODUCTION

### Problem Description

Prolonged immobility in critical care settings can lead to intensive care unit (ICU)–acquired weakness, affecting more than 25% of ICU survivors due to axonal nerve degeneration and muscle loss from systemic inflammation, medications, and electrolyte imbalances.^1–3^ Immobility significantly delays recovery and contributes to muscle atrophy. Children who experience critical illness can have a significant delay in returning to baseline function, and an early mobility program can help improve recovery time.^4^ Importantly, establishment of ICU-based mobility programs to implement mobility practices within 72 hours of admission decreased delirium, shortened length of stay (LOS), and improved emotional health without compromising safety.^5–9^

Infants with a neonatal intensive care unit (NICU) stay of more than 7 days are vulnerable to loss of muscle mass and chronic stress, which can impact neuronal development.^10–12^ In infants with respiratory failure, a 10% reduction or more in appendicular and diaphragm muscle thickness can occur within 5–7 days of mechanical ventilation.^11,13^

Former premature infants and infants with congenital anomalies face heightened risks of neurodevelopmental impairments or neurosensory disabilities.^14^ Although NICUs have long implemented developmental care programs such as the Neonatal Individualized Developmental Care and Assessment Program (NIDCAP) to support the mobility and development of premature infants, standard mobility guidelines for older, high-risk infants remain absent.^15^

NIDCAP is now the standard of care in the NICU for premature infants, and early enrollment is correlated with earlier discharge.^15^ NIDCAP primarily serves infants younger than 34 weeks’ postmenstrual age (PMA), leaving a care gap for older, critically ill infants. These patients, often managed at level IV NICUs, frequently experience disruptions in developmental care due to team changes and hospital transfers. Additionally, NICUs may lack trained personnel or infrastructure to support consistent developmental care.^16^ Therefore, a structured program is needed to promote seamless transitions and optimize developmental care for infants with advanced PMAs and prolonged critical illness.

The prevalence of disabilities among former PICU patients increased from 8% to 18% between 1982 and 2006, prompting the development of *PICU Up!*, a standardized, multicomponent early mobility program at the Johns Hopkins Hospital.^17,18^ The protocol included a standardized, evidence-based, interdisciplinary guideline to increase activity levels in critically ill children. The program successfully increased physical therapy (PT), occupational therapy (OT) consults and patient mobilization without adverse events and has been implemented and adapted in PICUs worldwide.^19^ Similar programs have also shown substantial increases in the percentage of appropriate PT and OT consults (92.5% and 91.6% respectively). However, barriers to optimizing therapy included staff apprehension about the safety of early mobility.^9^

To enhance developmental care for older infants, we sought to adapt and implement the early mobility *PICU Up! program* in a level III/IV NICU surgical population. We identified delays or absent PT/OT consults for infants 34 weeks’ PMA or older as a modifiable factor limiting early mobility. Our specific aim was to increase PT/OT consult rates for surgical patients 34 weeks’ PMA or older admitted to the Boston Children’s Hospital (BCH) NICU from a baseline of 25% and 22% to more than 75% within 24 months.

## METHODS

### Context

The BCH NICU is an academic quaternary referral center caring for outborn newborns with complex medical and surgical conditions. More than 650 infants are admitted each year, with approximately 150 infants admitted to the surgical team. Infants admitted to the surgical service are a complex population, most often referred for congenital or acquired anomalies requiring specialized surgical care, multidisciplinary management, and intensive perioperative support. All infants are cared for by a multidisciplinary team (NICU Staff) that includes neonatologists, general surgeons, neonatal and surgical fellows, neonatal nurse practitioners, neonatal nurses, nutritionists, lactation consultants, social workers, pharmacists, child life specialists, respiratory therapists, physical therapists, and occupational therapists.^19^

Before the study started, no consistent early mobility strategy existed for infants 34 weeks’ PMA or older, and there was no organized process for referral to PT and OT. At baseline, rounding providers and the nursing team decided whether to request PT and OT consults, and the individual providers placed consult orders in the Electronic Medical Record (EMR) Cerner PowerChart (Oracle Health, Kansas City, MO).

Johns Hopkins Children’s Center initially created and implemented the *PICU Up!* early mobility program. In 2018, BCH joined as a research site for *PICU Up!*, and in 2019, staff successfully implemented the program in both the pediatric cardiac and medical surgical ICUs. Due to unique considerations necessary to make the program appropriate for the NICU population, BCH did not implement the original *PICU Up!* in the NICU setting.

### Interventions

This quality improvement project focused on establishing a consistent early mobility program in the BCH NICU to increase PT/OT consult rates for infants admitted to the surgical service 34 weeks PMA or older and included a multidisciplinary study team.

### Needs Assessment and Key Driver Diagram Development

**Fig. 1.**
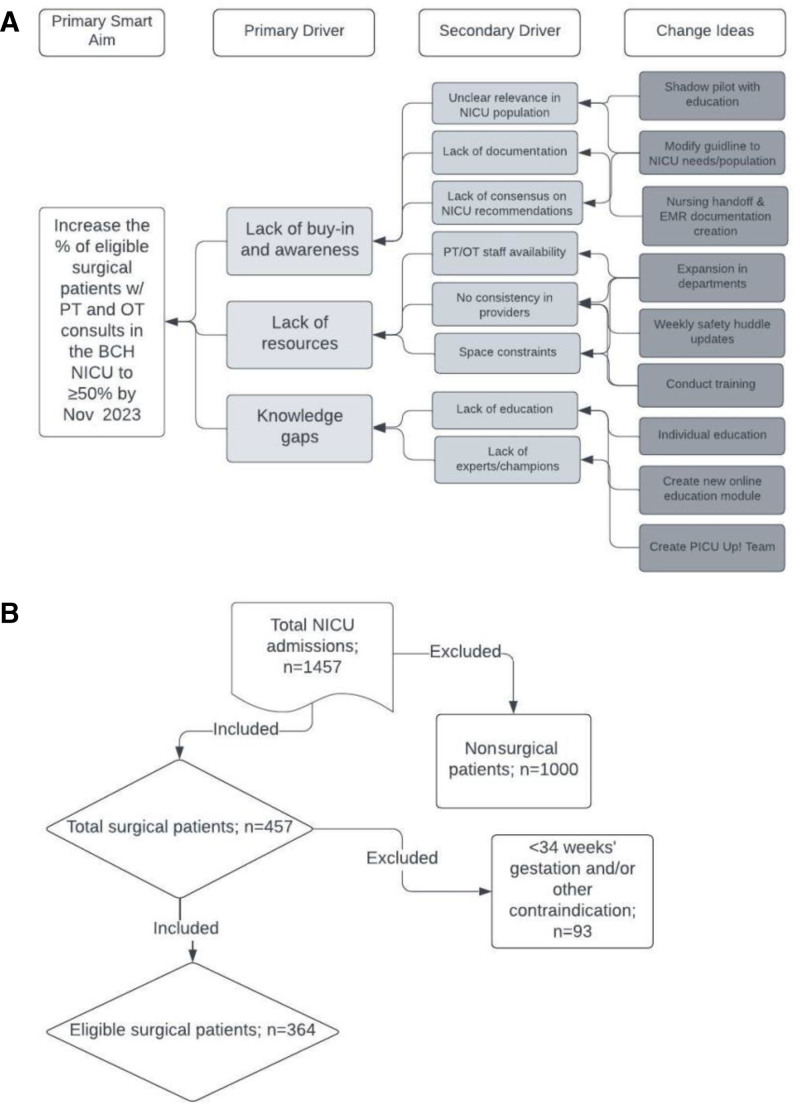
Key driver diagram and flow diagram of inclusion criteria. A, The key driver diagram outlines potential change concepts that affect secondary drivers, primary drivers, and primary project aims. B, Diagram of patient eligibility, including the total number of infants admitted and those excluded before analysis. For excluded infants in the surgical population, infants were ineligible if their gestational age at the time of PICU Up! assessment was less than 34 weeks’ gestation, if they had an open chest or abdomen, unstable fracture, medical orders specifying alternate activity, neuroprotective measures, or acute brain.

First, the study team conducted a multidisciplinary needs assessment, which identified top opportunities to improve the frequency of discussion, resource availability, and confidence in best practices for early mobility. Based on these identified gaps in developmental care, the team formulated a key driver diagram (Fig. 1A) and change ideas to test through multiple Plan-Do-Study-Act (PDSA) cycles.

### Modifying the PICU Up! Guideline to Be Applicable to Infant Populations

From August 2020 to November 2021, the team adapted the guideline to make it applicable, developmentally appropriate, and inclusive of the neonatal population (Table 1). Adaptations to the guideline included positive touch, positioning/mobility-specific activities for infants, clustering of care for infants younger than 6 months, no screen time, and the addition of safe sleep practices for eligible infants. Preterm infants were excluded from delirium screening, as no validated tool exists for this population. The team conducted 2 shadow pilots in January and March 2021 to test the adapted guideline in NICU infants and evaluate whether the PICU Up! levels and mobility goals were appropriate. The Johns Hopkins *PICU Up!* team reviewed and approved the adaptations in January 2022.

**Table 1.** Revisions to the *PICU Up!* Guideline^17^

PICU Up! Level		
Level 1	Level 2	Level 3
Patients: Critically ill, intubated, or sedated patients Focus: Physiological stability, sensory stimulation Activities: Passive ROM, repositioning, environmental enrichment, safe family interactions	Patients: Improving stability, minimal sedation Focus: Initiate active participation, upright positioning Activities: Sitting at the edge of the bed with support or supported sitting with caregiver; brief dangling or assisted trunk control exercises	Patients: Minimal support, active participation Focus: Weight-bearing, complex motor control Activities: OOB, supported standing or weight-bearing exercises, pivot transfers
Modifications to inclusion parameters		
Include patients with a new peritoneal dialysis catheter	Clarification to include patients on hemodialysis	
Includes patients on ECMO		
Modifications to activities		
Addition of positive touch	Addition of developmentally appropriate activities for infants when to OOB: holding/skin-to-skin care	Addition of activities for infants when OOB (chair, developmental positioning devices, holding for infants)
Addition of the “Reverse Trendelenburg for isolettes” to describe head of bed position	Addition of play, positioning, and positive touch	Addition of safe sleep practices
Addition of clustered care for infants <6 mo (bath, weight, repositioning)	Addition of communication challenges (new tracheostomy, prolonged intubation, tumor resection) as indications to consult augmentative communication/speech-language pathologist	
	Addition of AAP recommendations for limiting screen time in children <2 y: limit screen time or consult child life for alternatives	
	Exclusion of preterm infants in delirium screening	

The PICU Up! program uses a standardized algorithm to assign pediatric patients to one of 3 levels (levels 1–3) based on clinical status, with each level defining individualized daily mobility and rehabilitation goals. The first row of the table summarizes the patient characteristics, targeted goals, and permitted physical activities for each level. The second row details modifications to the original inclusion criteria to ensure applicability to NICU patients, presented by level. The third row outlines level-specific adaptations to the prescribed activities to accommodate the unique physiological and developmental needs of the NICU population.

AAP, american academy of pediatrics; ECM, extracorporeal membrane oxygenation; OOB, out of bed; ROM, range of motion.

### Education Rollout of the Adapted Neonatal PICU Up! Program for NICU Patients, System Changes, and Documentation

Initial interventions included the initial pilot launch of the adapted neonatal *PICU Up!* for the surgical population. The educational rollout of the adapted program consisted of multiple steps, including an introductory email sent in November 2021 as PDSA cycle 1. During PDSA cycle 2, NICU staff received personalized 1:1 education from a *PICU Up!* NICU champion, covering program goals, inclusion and exclusion criteria, level parameters, corresponding activity and mobility options for infants, and a demonstration of documentation procedures in the EMR. These recurring in-service sessions were held from November 2021 to March 2022. Educational one-on-one sessions also included case study reviews to apply the concepts learned during the training.

Next, PDSA cycle 3, from April to May 2022, was a systems and documentation enhancement that (1) added a *PICU Up!* level parameter field to the EMR and (2) added a *PICU Up!* section with level and mobility goals to the nursing written handoff sheet to enhance compliance with this new metric. The team distributed monthly emails to all unit staff and delivered presentations at unit and division meetings. They led education sessions for NICU nurse practitioners, attending physicians, and fellows to promote daily discussions during rounds.

PDSA cycle 4 was conducted from April to June 2022, which included online educational reinforcement and monthly communication to multidisciplinary staff about audit results. Additionally, they also refined EMR documentation.

### Study of the Interventions

The QI team established baseline data through a retrospective chart review spanning 21 months (January 2019–September 2020) to monitor outcomes and process measures in our NICU and assess the demographics of our population. The team collected prospective data for 24 months via manual weekly audits (November 2021–November 2023) to assess the impact of PDSA cycles on our measures over time. Audits included NICU admission date, PT and OT consult placement dates, *PICU Up!* level and goal on the nurse rounding sheet, ad PICU Up! level and goal in the EMR. Each month, the *PICU Up!* NICU champions reviewed weekly audits to determine whether new interventions were needed to boost compliance.

### Measures

Eligible infants included all patients admitted to the BCH NICU surgical service, regardless of operative status, and were 34 weeks' gestation PMA or older. Exclusion criteria were open chest, open abdomen, unstable fracture, medical orders specifying alternate activity, neuroprotective measures, or acute brain issues .

Process measures included (1) rate of PT consults (number of eligible patients with PT consults/total number of eligible patients); (2) rate of OT consultations (number of eligible patients with OT consults/total number of eligible patients); (3) time to PT (number of days from admission to first PT consultation); and (4) OT consultations (number of days from admission to first OT consultation). Outcome measures included (1) LOS (days from admission to transfer or discharge); (2) time to first extubation (TFE) (days); and (3) pressure injury rate (number of pressure injuries per month). Balancing measures included (1) rates of fractures (number of fractures per month), (2) rate of unplanned extubation (number of unplanned extubations per 100 ventilation days), and (3) safety events occurring during mobility activities. We also tracked the rate of compliance with documentation of level and assessment in the EMR.

### Analysis

The team used statistical process control charts to analyze data over time for key project measures. A substantial shift in the mean, as defined by control chart rules, indicated a significant change. P-charts and X-bar S-charts displayed the main measures, and the SQCpack software (PQ systems, Dayton, OH) generated the control charts. The team analyzed eligible infant demographic data in both pre- and postintervention periods using chi-square tests of independence, Mann–Whitney *U*, and Wilcoxon rank-sum tests to determine statistical differences, with statistical significance defined by a *P* value of less than 0.05. For the main outcome measures of TFE and LOS, the analysis compared the preintervention time period (from October 2020 to October 2021) to the postintervention period. Additional tests included a Mann–Whitney *U* test for nonpaired, non-normally distributed data and a Wilcoxon rank-sum test with continuity correction, using the same significance threshold (*P* < 0.05).

### Ethical Considerations

The institutional review board approved this project as exempt from human subjects research.

## RESULTS

### Needs Assessment

The multidisciplinary needs assessment survey revealed a strong consensus among NICU staff that early mobility improves outcomes, but it also identified several barriers. With a 30% response rate (n = 68), 63% of respondents reported a lack of confidence in their training on mobilization strategies, and 62% believed it would increase their workload. Half of the respondents cited insufficient resources, space, and patient categorization, whereas 62% reported inadequate discussion of mobility goals. Notably, all respondents agreed that patients were not too sick to be mobilized, 72% felt capable of identifying suitable candidates, and 69% believed families would want to participate. Additionally, 69% disagreed that PT and OT should be the sole providers of mobility care, expressing a strong desire for a team-based approach.

### Demographics

During the study period, 364 infants were eligible based on study criteria (Fig. 1B). There were no statistically significant differences among sex, race, birth weight, admission weights, and gestational age in the pre- and postimplementation stages. The overall median gestational age at birth was 36.3 weeks, with an interquartile range (IQR) of 3.9 weeks. The median birth weight was 3 kg with an IQR of 1, and the median admission weight was 3 kg with an IQR of 1. Fifty-three percent of patients were male. The median LOS was 7.99 days with an IQR of 17.07.

### Outcomes

**Fig. 2.**
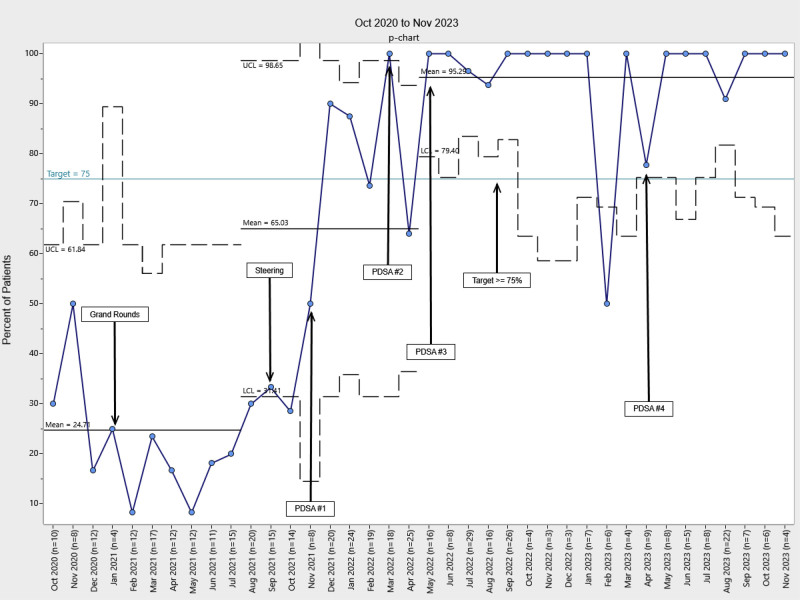
Percent of patients with PT consults. At baseline, 24.7% of patients completed a PT consult during their admission. There were 2 upward mean shifts during the intervention period: 1 started in August 2021, which increased to 65% (163.2% increase), followed by a shift in May 2022 to 95.3% (46.4% increase). LCL, lower control limit, UCL, upper control limit.

**Fig. 3.**
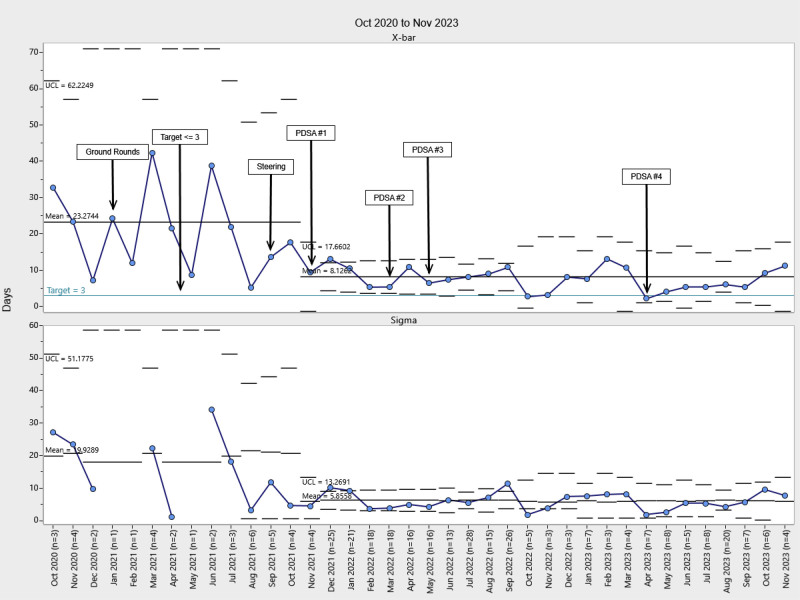
Time from NICU admission to PT consult. At baseline, the average time between admission and PT consult was 23.3 days. Starting in August 2021, the time decreased by 65.2% to 8.1 days and remained at that level throughout the intervention. UCL, upper control limit

**Fig. 4.**
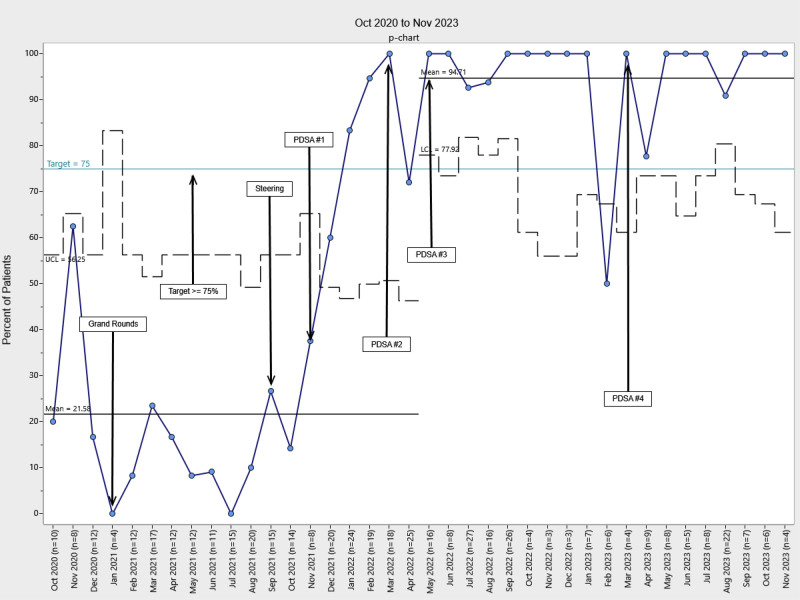
Percent of patients with OT consults. At baseline, 21.6% of patients completed an OT consult during their admission. Starting in May 2022, this percentage increased by 338.4% to 94.7% and remained at that level throughout the intervention. LCL, lower control limit, UCL, upper control limit.

**Fig. 5.**
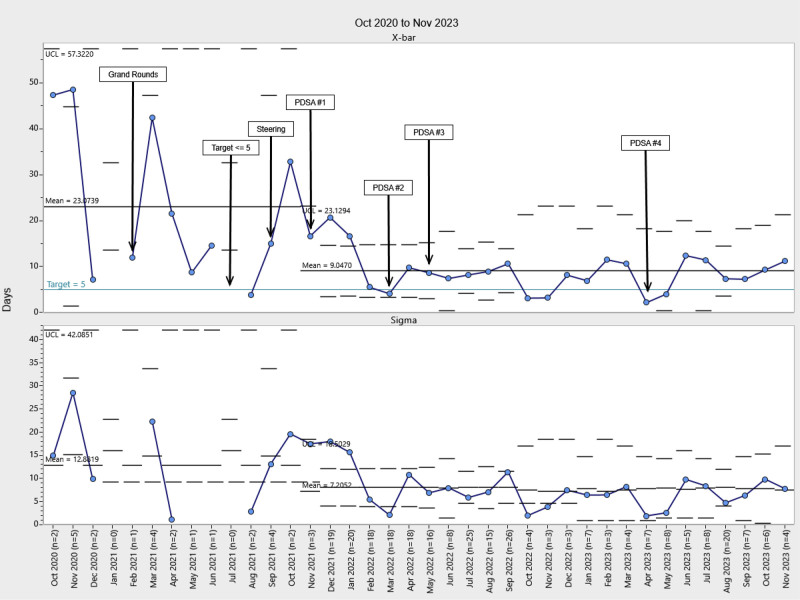
Time from NICU admission to OT consult. At baseline, the average time from admission to OT consult was 23.1 days. Starting in November 2021, the time decreased by 61% from 9 to 3 days and remained at that level throughout the intervention. UCL, upper control limit

Our chart audits revealed a high level of compliance with documentation and level assessments, with more than 75% documented in the EHR following the implementation of PDSA cycle 1. Control chart analysis revealed a significant increase in the percentage of patients with developmental care therapy consults following project implementation, with these consults occurring significantly earlier during the NICU stay (Figs. 2–5). Physical therapy consultations among surgical patients increased significantly, with two mean shifts demonstrating special cause variation. The first occurred in August 2021, after the program began, with a presentation to NICU leadership and the implementation of PDSA cycle 1. The second followed PDSA cycles 2 and 3 in May 2022. Following PDSA cycle 3, control chart analysis demonstrated a significant increase in the PT consultation rate from 25% to 95%, demonstrating high consistency and sustainability (Fig. 2). The timing of PT consultations also improved, decreasing from a baseline mean of 23 to 8 days between admission and consultation (a 65.2% reduction). This improvement persisted through the project period (Fig. 3). OT consults also increased significantly from a baseline of 22% to 95% of patients starting in May 2022 (PDSA cycle 3), with sustained improvement (Fig. 4). The timeliness of OT consultations improved, decreasing from 23 to 9 days after admission (a 61% reduction), and this improvement was maintained throughout the project period (Fig. 5).

For the main outcome measures, there was no significant differences between pre- and post-distributions for TFE (Wilcoxon rank-sum test, *W* = 2037.5, *P* = 0.7755) or for LOS (*W* = 11576, *P* = 0.7044). In the 8 months following the first PDSA cycle, the mean time from admission to first extubation in the BCH NICU decreased by 2.5 days, from a baseline of 6.9 to 4.4 days. In the 12 months following the first PDSA cycle, the median LOS in the BCH NICU decreased by 0.3 days, from a baseline of 7.4 days.

A Wilcoxon rank-sum test with continuity correction compared total ventilator days pre- and postimplementation and found that there was no significant difference in the number of vent days between the groups (*W* = 149.5, *P* = 0.6928). A 2-sample *t* test indicated no significant difference in the frequency of pressure injuries between the 2 groups (*t* = −0.80528, df = 31.186, *P* = 0.4268). Balancing measures demonstrated no significant difference in the frequency of fractures (*P* = 0.09) or unplanned extubations (*P* = 0.1141) between the 2 groups.

## DISCUSSION

Our team successfully adapted and implemented the *PICU Up!* early mobilization program for infants 34 weeks’ PMA or older in a level IV NICU, enhancing developmental care and bridging the gap for patients no longer eligible for NIDCAP. This structured, evidence-based approach ensured consistent developmental care across different types of ICUs, allowing patients and their families to have a reliable and steady approach to early mobility efforts, even if they transferred between units. Using a quality improvement framework to monitor data over time enabled our project team to test the effectiveness of efforts to overcome barriers to early mobility and focus on identified key drivers to meet the goals.

Implementation of the adapted *PICU Up!* program in the NICU surgical population included educational interventions, system changes, new practices, and enhanced team communication to increase staff buy-in. The guideline demonstrated feasibility and significant positive outcomes, with increased early mobility practices and no additional risks. The program led to key process improvements, including higher rates of PT/OT consultations and reduced time to consult initiation. By project completion, more than 94% of infants had PT and OT consults within 8–9 days of admission, overcoming prior barriers such as workload, resource limitations, and inconsistent practices. Increased NICU awareness of the importance of PT and OT services, improved use of available resources, and enhanced efficiency led to a greater focus on developmental care for infants who fall outside the traditional NICU developmental care programs. This study represented the first successful adaptation of an early mobility program for a neonatal population, specifically addressing the unique needs of surgical infants in a high-acuity, tertiary/quaternary care setting.

The postimplementation period showed no significant difference in the time to the first extubation attempt and NICU discharge. There was a trend noted in the reduction of mean time from admission to first extubation and a small reduction in the median LOS, which might suggest a direct impact of early mobility on short-term outcomes and developmental care in the postoperative patient. However, given significant variability in the data, the difference was not significant. Total ventilator days also remained unchanged. No significant changes occurred in pressure injuries, likely due to their already low incidence in the BCH NICU. The time to first activity of daily living (ADL) remained unchanged both before and after implementation, possibly due to the broad documentation criteria, highlighting a need for better-defined developmental activity tracking. Although ADL documentation differs between centers, it does not seem that documented ADL in our institution is an accurate way to measure time to first developmental activity. Importantly, no increased adverse events, such as fractures or unplanned extubations, occurred with the implementation of the guideline.

Documentation adherence audits and educational outreach to nursing staff sustained bedside engagement, as nurses documented the *PICU Up!* level and goal for each shift. This process measure ensured daily mobility discussions and consistent awareness of patient goals among the care team. After the project started, more than 75% of patients consistently had their *PICU Up!* level and goal documented in the EMR.

Incorporating a multidisciplinary approach to developmental care and recovery from complex illnesses or surgeries is essential in any critically ill population. Developmental programs in established NICUs often focus on earlier gestational ages, leaving late preterm to full-term infants without a structured approach to developmental care. Additionally, older infants often have short stays or are transferred to other units. This initiative successfully increased awareness and resource use to achieve the unified goal of increasing mobility in all NICU patients. The adapted *PICU Up!* guideline standardized mobility evaluations by integrating a structured, multidisciplinary approach into daily NICU practice, with the clinical team discussing mobility goals during rounds. Clearly defined criteria reduced subjectivity, fostering alignment among care teams and optimizing the use of PT/OT resources. Importantly, the entire care team participated in developmental care, focusing on early mobility. The initiative not only increased PT/OT consultations but also partnered with bedside nursing care to guide early mobility practices.

Limitations include the study’s single-center design and focus on a small target population of NICU surgical patients, limiting generalizability. However, there is likely high generalizability to the targeted NICU patient in this age group. Various elements of NICU care, along with other quality work and ongoing process improvements, may have affected the measures and influenced the outcomes. Broader validation across diverse neonatal populations and multiple centers is necessary to refine guidelines, assess long-term developmental impacts, and address potential biases in patient selection and care delivery. Our work focused on the timing and access to PT/OT consultations based on the timing of NICU admission. It could be helpful to focus on the postoperative period and maximize the mobility program in that specific segment of the NICU stay.

Additionally, future studies should investigate the program’s effect on more specific ADLs to more precisely assess its impact. Beyond the scope of this quality improvement intervention, further research to track the effects of early mobility practices on both short-term and long-term developmental outcomes could be of interest. Overall, strong multidisciplinary buy-in, improved outcomes, and the absence of adverse events indicate a successful, sustainable shift toward enhanced developmental care in the NICU.

## CONCLUSIONS

Adapting the *PICU Up!* program for the NICU using a standardized early-mobility framework was associated with increased PT and occupational therapy consultations, underscoring the value of coordinated, interdisciplinary care focused on early mobility. This quality improvement initiative highlights the essential role of developmental therapy services in the care of critically ill neonates and demonstrates the feasibility of implementing structured early-mobility programs in tertiary and quaternary NICUs, with potential applicability to similar units.

## ACKNOWLEDGMENTS

We appreciate the assistance of the NICU *PICU Up!* Championship Team in the implementation of this program: Jessica Herzog, NNP; Emily Serino, RN; Danielle Ouimet, RN; Sarah Cosford, RN; Jen McAvoy, RN; Heidi Tatelman, RN; Anna Olin, CCLS; Kayla Hernandez, MS, CCC-SLP; Coral Rudie, MS, RD; Emily Berry, PT, DPT; Chloe St. Rose, OT, MOT, OTR, CNT; Bethany O’Toole, PT, DPT, CNT, NTMTC; Phil Levy, MD.
